# Multiple Myeloma That Required Differentiation From Temporomandibular Joint Disorder: A Case Report

**DOI:** 10.1155/crid/4403039

**Published:** 2025-11-20

**Authors:** Kamichika Hayashi, Takaharu Ariizumi, Hiroshi Kato, Shuji Yoshida, Akira Watanabe

**Affiliations:** Department of Oral and Maxillofacial Surgery, Tokyo Dental College, Tokyo, Japan

**Keywords:** diffusion-weighted whole-body imaging with background body signal suppression, magnetic resonance imaging, mandibular condyle, multiple myeloma, temporomandibular joint

## Abstract

Multiple myeloma is characterized by a monoclonal neoplastic proliferation of plasma cells. This pathology can manifest as skeletal-related events (SREs), such as bone pain. However, such events are extremely rare in the mandibular condyle. We describe the case of an 82-year-old woman who presented to her dentist with occlusal pain in the right temporomandibular joint. Temporomandibular disorder (TMD) was diagnosed and treated using lifestyle modification. However, the pain did not subside, and she was referred to our department. On auscultation, crepitations were heard in the temporomandibular joint. Computed tomography showed an osteolytic lesion in the right mandibular condyle and cancellous bone loss in various locations. Abnormal ^18^F-fluorodeoxyglucose uptake was observed in the right mandibular condyle, among other locations. Blood testing revealed normocytic normochromic anemia, decreased renal function, delayed 1-h erythrocyte sedimentation rate, and decreased albumen and albumen-to-globulin ratio. Signal hyperintensity was evident in the right mandibular condyle on apparent diffusion coefficient mapping, while whole-body diffusion-weighted imaging with background body signal suppression revealed symmetrical diffusion-restricted areas in the right mandibular condyle, among other locations. These findings were diagnostic of multiple myeloma. The patient underwent chemotherapy, treatment for SREs, and oral care interventions to prevent medication-related osteonecrosis of the jaw (MRONJ). At present, the multiple myeloma remains stable, and TMD-like symptoms have disappeared. She can perform normal jaw movements, and no MRONJ has developed. Dentists must be aware that multiple myeloma can first present as orofacial manifestations. Care must be taken to avoid MRONJ when treating SREs of multiple myeloma.

## 1. Introduction

Multiple myeloma is characterized by plasma cell proliferation. The first manifestation of multiple myeloma varies; the disease may be asymptomatic or present as a skeletal-related event (SRE), such as bone pain associated with bone destruction. Frequent first manifestations include general malaise, anemia, hypercalcemia, renal dysfunction, and bone pain [1–5]. Multiple myeloma accounts for 1% of all cancers, 10% of hematological neoplasms, and 43% of malignant bone tumors. Most cases occur in patients over 60 years old, and a slight male predominance has been identified [6]. As the population ages, the prevalence of multiple myeloma is increasing each year. However, the disease presents with a diversity of clinical symptoms and will likely be encountered by an increasing number of medical staff across different departments. SREs are often observed in the vertebral, cranial, sternal, costal, iliac, and other long bones at the first examination. Observation of SREs in the mandible or maxilla is relatively rare, and SREs in the mandibular condyle are particularly rare [6–11].

We describe herein a rare case of multiple myeloma in which the first manifestation was observed in the temporomandibular joint region, and the diagnosis was reached using whole-body diffusion-weighted imaging (DWI) with background body signal suppression (DWIBS) [12].

## 2. Case Report

**Figure 1:**
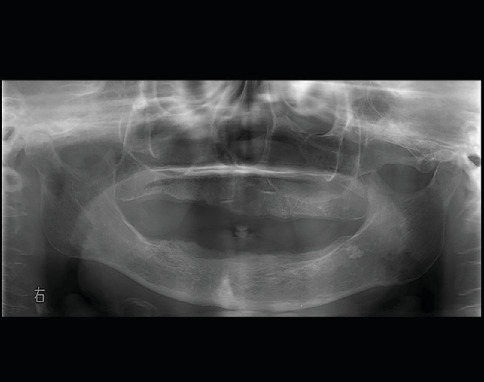
Panoramic radiography at first examination. No cysts, tumors, or structural changes are apparent in the maxilla or mandible.

**Figure 2:**
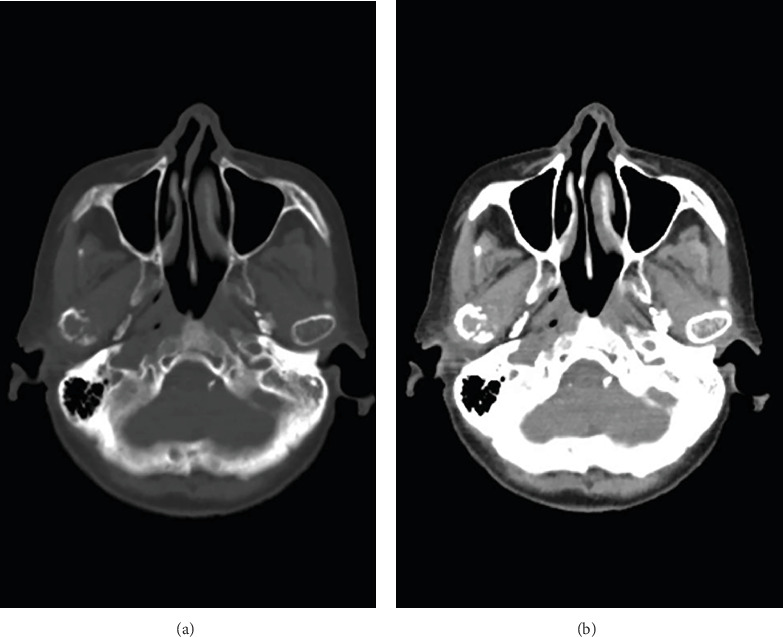
Axial computed tomography at first examination. (a) Hard tissue imaging. (b) Soft tissue imaging. An osteolytic lesion is observed in the right mandibular condyle.

**Figure 3:**
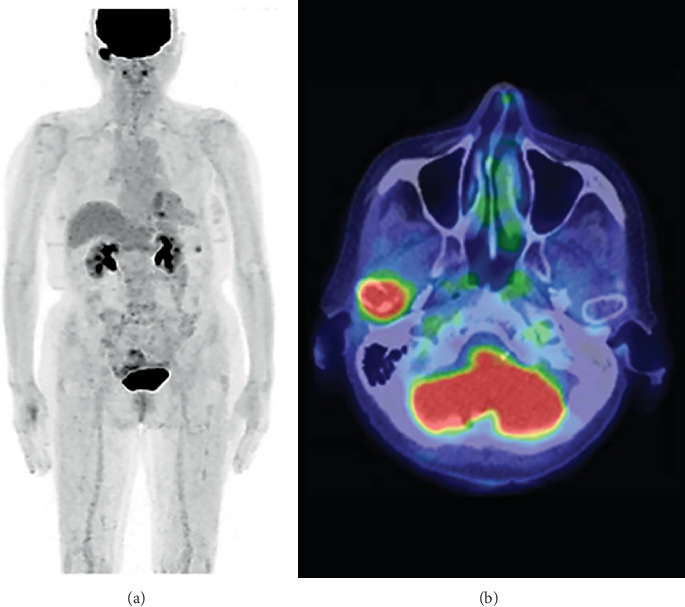
Results of ^18^F-fluorodeoxyglucose positron emission tomography and computed tomography (FDG-PET/CT). (a) Whole-body FDG-PET/CT. (b) Axial FDG-PET/CT. Maximum standardized uptake value was 7.62 in the right mandibular condyle, and abnormal FDG uptake was observed in the left eight rib and fourth thoracic vertebra.

**Figure 4:**
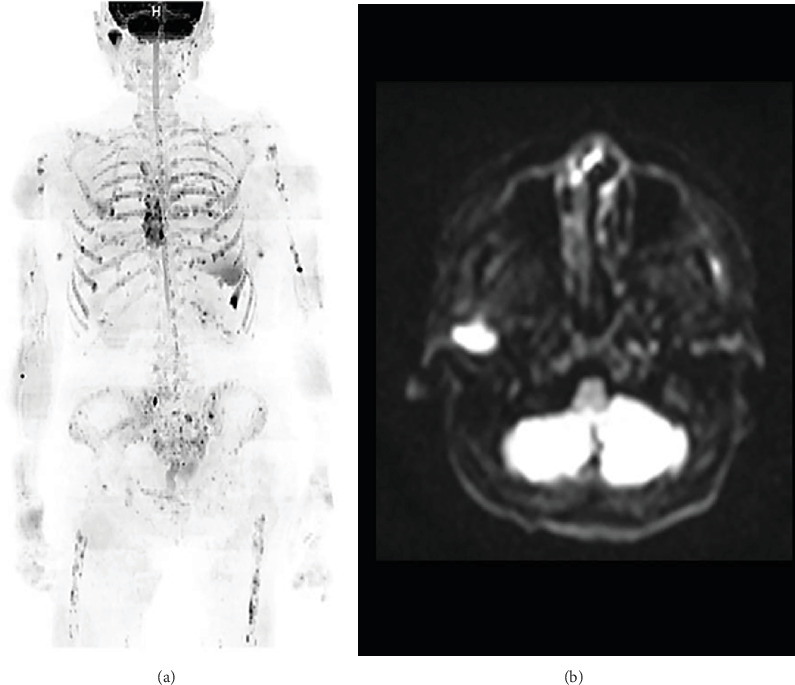
Magnetic resonance imaging (MRI). (a) On whole-body diffusion-weighted imaging with background body signal suppression, diffusion-restricted areas are observed systemically, including in the right mandibular condyle, sternum, ribs, humeri, femurs, and iliac bones. (b) Signal hyperintensity is observed in the right mandibular condyle on the apparent diffusion coefficient map of diffusion-weighted imaging.

The patient was an 82-year-old woman. She had suffered subjective symptoms of occlusal pain in the right temporomandibular joint region since August 2019. She visited a local dentist, who diagnosed right temporomandibular disorder (TMD). She was instructed to eat soft foods and to improve her tooth contacting habit (TCH). However, symptoms failed to improve, and she visited our department in September 2019 for detailed examination and treatment. Her chief complaint was pain in the right temporomandibular joint. She had no medical or family history of note. Body weight at first examination was 52 kg, and she had a lean body type. She had no fever but complained of general malaise. She was totally edentulous and wore full dentures on both jaws but had experienced no changes to occlusion. No evidence of intraoral infection was identified. Her face was symmetrical, with no evidence of swelling or redness. Bilateral translatory movements of the mandible were palpable, but crepitations were audible in the region of the right temporomandibular joint when the mouth was wide open. However, the patient did not complain of limited mouth opening. No pain was elicited on pressure to the masticatory muscles or, unexpectedly, on the painful part of the temporomandibular joint on either side. Panoramic radiography taken at the first examination showed no cysts, tumors, or structural changes in the maxilla or mandible (Figure 1). However, an osteolytic lesion was observed in the right mandibular condyle on computed tomography (CT) taken at the time of the first examination. Furthermore, the patient showed loss of the cancellous bone structures in the sternal, humeral, femoral, and iliac bones (Figure 2). Jaw bone metastasis of a malignancy was suspected, and ^18^F-fluorodeoxyglucose (FDG)-positron emission tomography (PET)/CT was performed. Maximum standardized uptake value was 7.62 in the right mandibular condyle, and abnormal FDG uptake was observed in the left eighth rib and fourth thoracic vertebra (Figure 3). Blood testing showed normocytic normochromic anemia, decreased red blood cell count, decreased platelet count, decreased white blood cell count, decreased renal function, a delayed 1-h erythrocyte sedimentation rate, and decreased albumen and albumen-to-globulin ratio (Table 1). Based on all these findings, multiple myeloma was suspected, and additional magnetic resonance imaging (MRI) was performed. High signals were observed in the right mandibular condyle on the apparent diffusion coefficient (ADC) map from DWI. On DWIBS, diffusion-restricted areas were observed systemically, such as in the right mandibular condyle, sternum, ribs, humeri, femurs, and iliac bones (Figure 4). Based on all these findings, multiple myeloma was clinically diagnosed.

**Figure 5:**
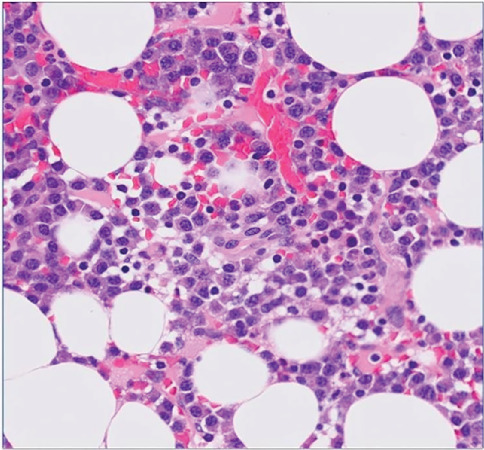
Histopathological analysis. Hematoxylin and eosin staining; original magnification, ×400. Bone marrow biopsy from the iliac bone shows 30% cellularity and interstitial infiltration by plasma cells.

The TMD-like symptoms in the region of the right temporomandibular joint may have been linked to SREs of multiple myeloma in the right mandibular condyle, so the department of hematology and oncology was consulted, where additional blood and urine tests were performed (Table 2). We further checked the level of free light chains (FLCs) *κ* and *λ* immunoglobulin (Ig) light chains by blood test (FLC-*κ*: 55.3 mg/L, FLC-*λ*: 2.75 mg/L, and FLC-*κ*/FLC-*λ*: 20.109). Furthermore, a hematology and oncology specialist obtained a marrow biopsy from the iliac bone (Figure 5). Pathological analysis of the marrow specimen showed 30% cellularity, Congo-red negativity, direct fast scarlet negativity, and > 80% CD138-positive cells. The pathological diagnosis was Ig G Type *κ* multiple myeloma, classified as Stage II in the International Staging System and Stage III in the Durie–Salmon staging system [13–15].

In the department of medical oncology, chemotherapy was performed for primary multiple myeloma not indicated for autologous peripheral blood stem cell transplantation. First, VRd (bortezomib + lenalidomide + dexamethasone) was administered for two cycles, followed by D-VMP (daratumumab + bortezomib + melphalan + prednisolone) for one cycle, with a subsequent switch to BD (bortezomib + dexamethasone) to continue chemotherapy.

In our department, TMD-like symptoms of the right temporomandibular joint region were diagnosed as bone pain associated with SREs from multiple myeloma. Denosumab was initiated to treat the SRE. To prevent bone pain or pathological fracture, we provided lifestyle education, correction of TCH, and diet-related guidance, such as instructions to select soft solid and liquid foods. We chose this approach over surgical treatment or radiotherapy in the right mandibular condyle to prioritize patient quality of life (QOL). Moreover, oral care interventions were implemented to prevent medication-related osteonecrosis of the jaw (MRONJ) [16].

Although remission of the multiple myeloma has not been achieved as of the time of writing, the patient has been followed up without exacerbation. No bone formation has been observed in the right mandibular condyle, but TMD-like symptoms have disappeared and the patient can perform physiological jaw movements without functional disorders such as adhesion. No MRONJ has been observed and QOL has been maintained for the patient.

Fully informed consent for publication of clinical information relating to this case was obtained from the patient.

## 3. Discussion

Multiple myeloma is a primary bone tumor characterized by monoclonal neoplastic proliferation of plasma cells. The disease is diagnosed based on the presence of abnormal monoclonal plasma cells and the results of a full blood count, bone marrow biopsy, IgM-protein levels in the serum or urine, and clinical images [7]. The pathology accounts for approximately 1% of all malignancies and approximately 10% of hematological malignancies and is most common among men in their 50s–80s [6–9, 17]. The clinical manifestations are caused by the expanding mass of plasma cells in the bone marrow, and common clinical symptoms include bone pain (upper and lower back pain), malaise, fever, anemia, hypercalcemia, renal failure, and infections [1–5]. Only rarely do symptoms occur in the stomatognathic system, with facial and gingival swelling as the most common such symptom, followed by dental mobility, gingival bleeding and inflammation, sensory disturbance, jaw pain, and pathological fracture [7, 9, 17]. Having the first manifestation of multiple myeloma occur in the stomatognathic area is extremely rare [7, 8, 18–20], especially in the temporomandibular joint region [6–11, 21]. Moreover, the clinical symptoms of multiple myeloma in the temporomandibular joint region are TMD-like and nonspecific, which is likely why they are often misdiagnosed as TMD.

Manifestations of multiple myeloma in the stomatognathic region can be distinguished from differential diagnoses such as odontogenic tumors and cysts, arteriovenous malformations, carcinomas of the maxillary sinus or gingiva, central carcinoma of the jaw bone, osteosarcoma, and cancer metastases from other organs based on the characteristic punched-out lesions on radiography [8, 9, 17].

Our patient was clinically diagnosed with multiple myeloma based on her advanced age, malaise, anemia, decreased renal function on laboratory blood tests, osteolytic lesions on CT, abnormal FDG uptake in the right mandibular condyle, left eight rib and thoracic vertebra, and multiple bone marrow lesions and extramedullary lesions evident on MRI.

MRI shows superior contrast resolution of soft tissues and is suitable for visualizing lesions of the bone, marrow, joints, and soft tissues. Furthermore, this modality is superior to bone scintigraphy or FDG-PET/CT for visualizing bone marrow lesions [22–26]. DWIBS involves no exposure to radiation, is noninvasive because no contrast agents are required, and is superior for imaging the entire body from bone and marrow lesions to solid organs [27–29]. In 2015, the International Myeloma Working Group recommended whole-body MRI as the gold standard test for bone lesions to diagnose multiple myeloma [25]. Papers are beginning to be published showing that MRI is superior to FDG-PET/CT in diagnosing multiple myeloma [24, 25, 30]. Therefore, we performed an additional MRI to evaluate the effectiveness of future treatment. Indeed, DWIBS was also more effective than FDG-PET/CT for diagnosing multiple myeloma in our patient because marrow and extramedullary lesions are better visualized. However, few facilities are currently equipped with DWIBS in Japan. Accumulation of more data on multiple myeloma cases diagnosed using DWIBS is warranted.

SREs of multiple myeloma can dramatically lower patient QOL and activities of daily living. Bone-modifying agents (BMAs) such as bisphosphonate and denosumab are the gold-standard first-line therapies for SREs in multiple myeloma, as they can alleviate bone pain, prevent pathological fractures and other SREs, and prolong overall survival [1, 5, 9, 16]. However, MRONJ has been reported in patients receiving BMA therapy and markedly decreases patient QOL. Dentists play an essential role in providing routine oral care to prevent MRONJ [8, 31–33].

As the population ages, more patients with multiple myeloma are expected to consult a dentist first, so multiple myeloma should be considered when responding to patients of advanced age seeking dental care for TMD symptoms combined with malaise, anemia, and decreased renal function.

We have reported a rare case of multiple myeloma presenting with pain in the mandibular condyle as a first sign. We also described how DWIBS is useful in diagnosing multiple myeloma. This disease can present with a variety of orofacial manifestations. Dentists should properly address oral manifestations as first indications of multiple myeloma. The SREs of multiple myeloma may respond better to BMAs, but care must still be taken to avoid MRONJ.

## Figures and Tables

**Table 1:** Blood testing showed normocytic normochromic anemia, decreased renal function, a delayed 1-h erythrocyte sedimentation rate, and a decreased albumen and albumen-to-globulin ratio.

WBC	3700	/*μ*L
RBC	2,540,000	/*μ*L
Hb	8.7	g/dL
Ht	26.4	%
Plt	124,000	/*μ*L
TP	11.6	g/dL
Alb	3.2	g/dL
A/G ratio	0.38	
AST	49	U/L
ALT	17	U/L
*γ*-GPT	11	U/L
ALP	165	U/L
BUN	19	mg/dL
Cr	1.08	mg/dL
Na	135	mEq/L
Ca	9	mEq/L
Cl	103	mEq/L
K	3.8	mEq/L
CRP	0.04	mg/dl
ESR	134	mm/1 h

**Table 2:** The department of hematology and oncology was consulted, where additional blood and urine tests were performed. These indicated the possibility of multiple myeloma IgG-K type.

IgA	22	mg/dL
IgG	6428	mg/dL
IgM	6	mg/dL
F-LC・к/*λ*	20.109	
F-LC-к	55.3	mg/L
F-LC-*λ*	2.75	mg/L
*β*2MG	5.12	mg/L
Bence Jones protein	Negativity	

## Data Availability

The data that support the findings of this study are available from the corresponding author upon reasonable request.
